# Facilitators of Nursing Assistant Job Commitment and Career Retention in VHA Community Living Centers

**DOI:** 10.1093/geroni/igaf122.2142

**Published:** 2025-12-31

**Authors:** Katherine Kennedy, Nathalie Mcintosh, Sylvia Haigh, David Mohr, Ciaran Phibbs, Whitney Mills

**Affiliations:** Providence VA Healthcare System, Providence, Rhode Island, United States; THRIVE, Providence VA Medical Center, Providence, Rhode Island, United States; VA Providence Healthcare System, Providence, Rhode Island, United States; Veterans Health Administration, Mason, Ohio, United States; Health Economics Resource Center, VA Palo Alto Health Care System, Menlo Park, California, United States; Brown University, Providence, Rhode Island, United States

## Abstract

Job satisfaction matters for the retention of long-term care aides; however, we know little about this in the Veterans Health Administration (VHA) setting. This study examined the facilitators and barriers to NA job commitment in VHA Community Living Centers (CLCs; VHA nursing homes). We recruited 4 CLCs and interviewed 6-9 staff at each (N = 30), of which 17 were NAs and 13 were nurse leaders. We employed qualitative content analysis based on codes relating to Paraprofesional Healthcare Institute’s (PHI) Five Pillars of Direct Care Job Quality. 100% (N = 17/17) of NAs reported intention to remain employed at the CLC in the next 12 months and/or reported high job satisfaction. Interviewees reported many factors related to job commitment and satisfaction. Facilitators included VA benefits (e.g., healthcare, retirement), passion for NA work, relationships with the Veterans, favorable work conditions (e.g., workload, safe patient handling equipment, training, steady work), and a supportive work environment (e.g., supportive managers/coworkers, teamwork). Suggestions for improvement in retention included addressing dissatisfaction with pay (e.g., non-competitive, pay caps), scheduling, career advancement, staffing (e.g., shortage, human resource (HR) challenges), management practices (e.g., mandatory overtime, inability to take requested leave, floating), staff- and resident-to-staff interactions (e.g., disrespect), and structural supports (e.g., no onsite HR). Job commitment and satisfaction are multilayered. Despite desiring certain changes, NAs remain committed to their roles. VHA nursing home care has been affected by general nursing workforce shortages. Promoting policy change related to NA wages and pay caps concurrent with supportive training, scheduling, and employee wellness may improve retention.

